# Bullying and Cyberbullying in School: Rapid Review on the Roles of Gratitude, Forgiveness, and Self-Regulation

**DOI:** 10.3390/ijerph21070839

**Published:** 2024-06-27

**Authors:** Wanderlei Abadio de Oliveira, Antonio Marcos Neves Esteca, Solange Muglia Wechsler, Ersilia Menesini

**Affiliations:** 1 Graduate Program in Psychology, School of Life Sciences, Pontifical Catholic University of Campinas, Avenida John Boyd Dunlop, Jardim Ipaussurama, Campinas 13060904, SP, Brazil; tecnologia@faculdademetropolitana.edu.br (A.M.N.E.); wechsler@puc-campinas.edu.br (S.M.W.); 2 Department of Education, Languages, Intercultures, Literatures and Psychology, University of Florence, Via San Salvi 12, Padiglione 26, 50135 Florence, Italy; ersilia.menesini@unifi.it

**Keywords:** bullying, character strengths, cyberbullying, positive psychology, systematic review

## Abstract

This study aims to assist decision-making in anti-bullying interventions by highlighting the importance of positive factors such as gratitude, forgiveness, and self-regulation in mitigating the negative impacts of bullying/cyberbullying. The objective was to examine and synthesize available evidence on the impact of gratitude, forgiveness, and self-regulation practices in the school context regarding bullying/cyberbullying phenomena. Three databases were consulted (Web of Science, Scopus, and Scielo), and the results include 14 articles. The three character strengths were associated with psychological well-being, life and school satisfaction, improved mental health, increased likelihood of engaging in pro-social behavior, and reduced involvement in bullying/cyberbullying situations. These strengths have the potential to enhance overall well-being and decrease risk behaviors, leading to more positive outcomes in experiences of violence. These results underscore the importance of considering students’ individual strengths and the possible interventions to promote healthy school environments.

## 1. Introduction

Adolescence is a developmental phase characterized by various biological, behavioral, and psychosocial changes. It can be a tumultuous and confusing period in a person’s life, where contradictory and inconsistent feelings may manifest as aggressive behaviors, such as school bullying or cyberbullying. Bullying involves the repeated and intentional expression of aggressive behaviors in peer relationships based on a power imbalance between victims and aggressors [1,2]. With increased access to the internet, the phenomenon has shifted to the online realm, and is now referred to in this space as cyberbullying [1]. It is observed that many cyberbullying victims are also victims of traditional bullying, indicating that cyberbullying creates additional victims [1].

According to the National School Health Survey (*Pesquisa Nacional de Saúde do Escolar*), 2019 edition, in Brazil, approximately 12% of students engage in bullying at school, and 23% are victims [3]. Regarding the prevalence of cyberbullying, around 13% of students feel threatened, offended, or humiliated on social media or on applications on their cell phones [3]. The consequences of bullying and cyberbullying can be categorized into three main areas: educational consequences, health consequences, and consequences that extend into adulthood [4].

Recent studies suggest that positive personality traits, or character strengths, may play a crucial role in mitigating the negative impacts of bullying and cyberbullying. These traits are thought to foster pro-social behavior and reduce involvement in such negative activities. However, research on the topic (bullying and cyberbullying) has heavily focused on diagnosing the phenomena and seeking associations with psychosocial variables. The association between non-cognitive and cognitive personality traits and bullying/cyberbullying has not been a research topic. In this regard, especially positive and desirable personality traits may be associated with higher levels of pro-social engagement or non-involvement in bullying/cyberbullying situations. It is in this direction that this review focuses on three character strengths to analyze the issues of bullying and cyberbullying.

Character strengths are positive personality traits that help individuals engage in morally valued behaviors [5]. They are considered “values in action”, as each strength is related to the application of a specific virtue and reflects the psychological mechanisms that promote its practice. Everyday life is facilitated by the expression of character strengths through behaviors that impact people’s social experiences [6]. There are a total of 24 character strengths grouped into 6 essential virtues: (1) creativity, curiosity, judgment, love of learning, and perspective (virtue of wisdom and knowledge); (2) bravery, perseverance, honesty, and enthusiasm (virtue of courage); (3) love, kindness, and social intelligence (virtue of humanity); (4) teamwork, justice, and leadership (virtue of justice); (5) forgiveness, humility, prudence, and self-regulation (virtue of temperance); (6) appreciation of beauty and excellence, gratitude, hope, humor, and spirituality (virtue of transcendence) [7].

Gratitude and forgiveness, specifically, are interpersonal strengths that promote well-being, happiness, and increased pro-social behavior by eliciting a combination of reflection, positive emotions, adaptive behaviors, and social relationships. These strengths share empathy as a common psychological component [6,8]. Individuals reporting higher levels of gratitude and forgiveness tend to report less anger and feelings of loneliness, as well as fewer depressive symptoms [5,6,9]. These individuals also report greater acceptance, empathy, and self-compassion. Self-regulation, in turn, is associated with healthier lifestyles and a reduction in adopting risky behaviors [5]. The selection of these three character strengths was based on their relevance in promoting positive behaviors and reducing involvement in bullying and cyberbullying.

While many studies have explored protective factors in bullying behavior, including forgiveness, gratitude, and self-regulation, there is still a need to synthesize and highlight these aspects comprehensively. We aim to examine and synthesize available evidence on gratitude, forgiveness, and self-regulation practices in the school context regarding bullying/cyberbullying phenomena. The hypothesis to be tested in this review considers that the presence and development of specific personality traits (gratitude, forgiveness, and self-regulation) may be associated with a decrease in involvement in bullying or cyberbullying situations. As this is an exploratory review, no specific roles of students (e.g., victims, aggressors, or bystanders) or the effects of these characteristics on one role or another are particularized. It is expected that a literature review will describe how these aspects are studied and the general understanding related to these factors.

## 2. Materials and Methods

### 2.1. Study Type

This is a rapid review characterized by the application of an accelerated process in the search and synthesis of knowledge on a specific topic [10]. This type of review is no less systematic than other types, and is useful for gathering evidence to facilitate primary clinical decision-making. The Cochrane guidelines for conducting rapid reviews were followed [11]. In this review, the following steps were applied: definition of the research question; definition of eligibility criteria (including time limitations to be considered and the number of databases to be consulted); search, selection, and data extraction; and data analysis and the construction of interpretative synthesis [10,11].

### 2.2. Guiding Question

To construct the guiding question for the review, the PCC strategy (population, concept, and context) was used [12]: How do character strengths such as gratitude, forgiveness, and self-regulation in adolescents interact or influence the dynamics of bullying/cyberbullying in school settings?

### 2.3. Search Strategies

The search was conducted in three databases: Web of Science, Scopus, and Scielo. Search terms and cross-references were developed to capture publications that addressed the proposed objective: gratitude AND bullying; forgiveness AND bullying; self-regulation AND bullying. The terms were also used in Portuguese on Scielo. The strategies employed in each database are described in Table 1.

The results were exported to the Rayyan platform [13]. Initially, each abstract and title were evaluated by one researcher who selected items to read in full. Full texts were also assessed by a researcher. Another researcher independently supervised and guided the corpus selection process. Data extraction was conducted by the two researchers, who regularly met to address concerns and ensure that data extraction was consistently carried out following the review’s objective and guiding question, as well as the application of inclusion and exclusion criteria.

### 2.4. Inclusion and Exclusion Criteria

The review covered the last five years (2019–2023). This time limit was defined to reach the most current scientific literature and to follow the guidelines for conducting this type of review. Articles reporting empirical research were eligible if they focused on children and adolescents, addressing issues related to bullying or cyberbullying in schools. Only texts published in English, Spanish, or Portuguese were considered. Articles were excluded if they involved other populations (children, young adults, or adults, for example); focused solely on broad contextual factors related to the investigated phenomena; were opinion articles, protocols, or reviews; or concentrated on the conception or evaluation of instruments or interventions.

### 2.5. Data Analysis

The descriptive analysis involved systematically summarizing the key findings from each study. This included quantifying the frequency of certain variables and outcomes, categorizing the types of bullying and cyberbullying behaviors observed, and noting the prevalence of positive character strengths. The exploratory analysis aimed to identify and examine underlying patterns and relationships within the data that might not have been immediately evident through descriptive methods alone. This involved a more in-depth examination of the interactions between different variables, such as how gratitude, forgiveness, and self-regulation correlated with bullying and cyberbullying behaviors. Following the descriptive and exploratory analyses, an interpretative synthesis was created. This synthesis involved integrating the findings from the individual studies into a coherent narrative that emphasized the most important conclusions. The researchers critically evaluated the evidence and discussed its implications for understanding the role of positive character strengths in addressing bullying and cyberbullying.

## 3. Results

### 3.1. Constitution and Characteristics of the Reviewed Corpus

**Figure 1:**
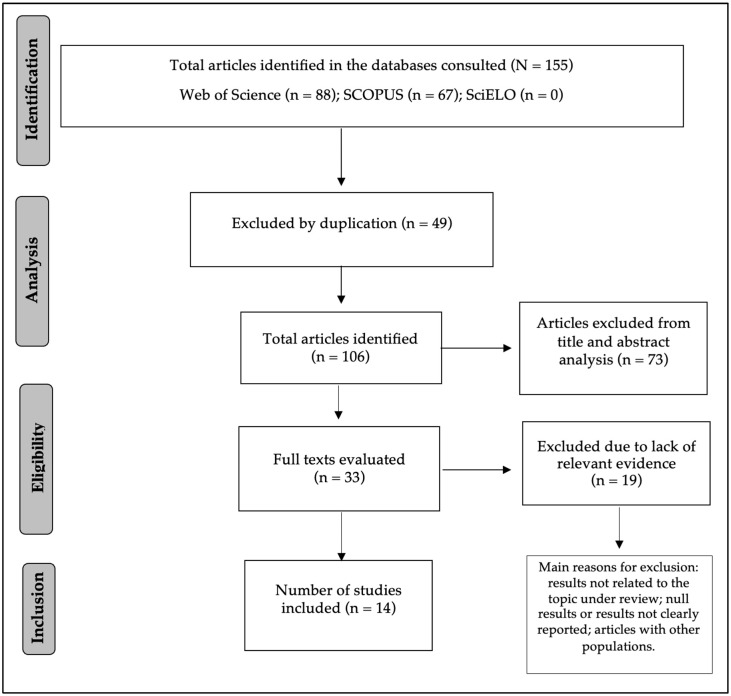
Diagram of the search process and selection of articles for the review (PRISMA).

The initial screening of titles and abstracts was conducted on the Rayyan platform, excluding duplicates (*n* = 49) and studies with results not pertinent to the review or involving other populations (*n* = 73) in the first instance. Thirty-three articles were selected in the final corpus selection to be read in full. The selection process is detailed in the PRISMA flowchart available in Figure 1.

Five studies were conducted in Spain, two in China, two in Peru, and two in Mexico. Australia, Italy, and Turkey each contributed one study, and no research conducted in Brazil was found. Among the included studies, 13 were cross-sectional, and 1 had a longitudinal nature [14]. The smallest recorded sample consisted of 43 adolescents, while the largest sample was from a study with 2.758 participants. Ages varied between 9 and 19 years in the studies. A variety of instruments were used to collect data, mainly to assess bullying or cyberbullying situations. Table 2 presents the descriptive data of the reviewed corpus.

It was observed that gratitude was a variable present in various studies, often associated with other variables such as cyberbullying, mindfulness, life satisfaction, compassion, moral development, and pro-social behavior. Forgiveness was also a common variable frequently studied in relation to cyberbullying, well-being, self-control, revenge motivations, self-esteem, stress, and bullying. Among the variables of interest in the review, self-regulation—from the perspective of positive psychology—was the least identified. This initial analysis demonstrates that there is a diverse range of variables that can interact with important concepts for understanding the dynamics of bullying/cyberbullying, but further studies on the relationship with self-regulation are still needed.

### 3.2. Primary Outcomes Reported in the Studies

Considerable rates of victimization and the perpetration of bullying or cyberbullying were revealed. One of the included studies, for example, identified a rate of involvement in bullying situations of approximately 35% [22]. Another study identified cybervictimization rates of 23% and cyberbullying practices of approximately 18% in a final sample composed of 979 Spanish adolescents [23]. The results of the reviewed studies explore a wide range of factors related to bullying and cyberbullying, as well as the relationships established between these phenomena and gratitude, forgiveness, and self-regulation. It was found that, in many studies, character strengths act as mediators for other issues such as mental health frameworks, or the emission of pro-victim behaviors, for example. 

A separation between bullying and cyberbullying data is necessary to better understand the specifics of the included studies. In this regard, it is observed that six studies presented data on bullying [18,19,20,21,22,24]. Students who observe bullying situations in schools, for example, and exhibit feelings of gratitude, forgiveness, compassion, or happiness tend to display more pro-social behaviors [18,22]. One study also found that adolescents who have high levels of forgiveness, gratitude, and self-control are less likely to engage in bullying situations [19]. This occurs because these characteristics help manage negative emotions and promote more positive and constructive responses to conflicts and challenges. On the other hand, students with high levels of victimization showed a greater desire for revenge, greater motivation for school avoidance, feelings of loneliness, and a more negative evaluation of their support networks [20]. Students victimized in schools also tended to have fewer feelings of gratitude [24]. Eight studies presented data on cyberbullying [14,15,16,17,23,25,26,27]. In these cases, students who reported being able to forgive or who had effective strategies for dealing with cyberbullying showed higher well-being despite the victimization they suffered [17]. Cybervictimization was also significantly associated with stress and the motivation for revenge, as well as a lower availability to exercise forgiveness [23,25]. Other results can be checked in detail, study by study, in Table 2.

Regarding gratitude, in general, it was demonstrated to play a significant role in promoting emotional well-being and combating the negative effects of bullying/cyberbullying. Additionally, gratitude correlated positively with pro-social behavior towards victims and acted as a mediator in the relationship between dimensions of emotional intelligence and cyberaggression, partially or fully explaining how these dimensions affect aggressive behavior. In this regard, adolescents who were victims of bullying, especially cyberbullying, were more likely to exhibit symptoms of depression. However, feeling gratitude was associated with fewer depressive symptoms among victims, especially girls. On the other hand, adolescents more sensitive to acts of kindness tended to be less involved in bullying behaviors.

It was evidenced that forgiveness was related to reduced levels of depression, lower involvement in revenge behaviors, and a lower likelihood of participation in bullying or cyberbullying situations. Furthermore, it was identified that the willingness to forgive positively influenced the emotional well-being and self-esteem of adolescents, even in cases of victimization. Forgiveness also had a significant impact on the experiences of bullying victims, influencing the desire for revenge, emotional loneliness, and perceptions of social networks.

Regarding self-regulation, it was observed that students who rarely expressed guilt and sympathy in cyberbullying events demonstrated moderate self-regulation. Moreover, being female was positively related to self-regulation, while the negative correlation between self-regulation and aggressive defensive behavior suggests that the better someone is at self-regulating their emotions and actions, the less likely they are to adopt an aggressive intervention when witnessing cyberbullying.

Another necessary separation in the data analysis concerns the role of students (victims, aggressors, or bystanders) in bullying or cyberbullying situations. In this regard, victims who reported being able to forgive or who had effective strategies for dealing with cyberbullying showed greater well-being despite the victimization suffered [17]. For adolescents with a low ability to express forgiveness, victimization had a significantly negative impact on subjective well-being and mental health [20,21,23]. In Italy, cybervictimized boys were less willing to forgive [25]. Victimized students in Spanish schools also tended to have fewer feelings of gratitude [24]. Regarding aggressive behaviors, studies reported that students with higher levels of forgiveness, gratitude, and self-regulation were less likely to engage in bullying or cyberbullying situations [16,19,23,25]. For bystanders, it was also found that pro-social behaviors towards victims were associated with the ability to express feelings of gratitude, forgiveness, or compassion [18,22].

Some key results on gender differences should also be explicitly stated, even though this was not one of the purposes of this review. In this regard, overall, girls in the various studies reported possessing more positive aspects than boys (gratitude, forgiveness, and self-control, for example). For instance, one study found that, in general, girls tend to report higher levels of gratitude, forgiveness, happiness, and pro-social behavior when witnessing bullying situations in schools [18]. Generally, boys were more aggressive and had less gratitude than girls [19]. Boys who were victims of cyberbullying also showed less willingness to forgive [25]. Additionally, boys who were able to forgive considered aggressive events less serious over time [27]. Additional studies are recommended to better understand this dynamic from a gender perspective.

Additionally, in the analysis of these studies, it was verified how the studies ensured the validity and accuracy of the results. In this sense, it is observed that, as the studies mostly had a cross-sectional nature, the validity and accuracy of the results depend on various statistical practices and techniques. The researchers adopted appropriate statistical practices to assess the validity and accuracy of the results. They used a variety of statistical methods, including tests, correlation analyses, and modeling, while controlling for confounding variables and adopting a specific statistical significance level to assess the significance of the results (information contained in the Supplementary Materials). These practices are fundamental for conducting valid and reliable research. It is also noted that Table 2 describes the main results revealed in each study. 

### 3.3. Interpretative Synthesis

This review presents a solid foundation on students’ experiences of bullying and cyberbullying. Additionally, empirical data provide a relevant framework for understanding how values in action or character strengths in the individual field can, in terms of mental health primarily, change the outcomes of these experiences. It can be considered that this review adds information about phenomena that affect adolescent development and can be used in clinical interventions or within school contexts. It is also noted that the reviewed data are from local realities, but with social and experiential foundations that can be considered globally.

Specifically, regarding the guiding question of this study, it was noticed that gratitude has been explored as a potential factor to improve the quality of life and psychological state of students involved in bullying situations, especially those who are victimized either traditionally in school or online. Promoting the feeling of gratitude was also considered a valuable approach for preventing violence phenomena among adolescents. Regarding forgiveness, the studies revealed a lack of initiatives to help students use this feeling as a coping mechanism for bullying/cyberbullying or to improve well-being. Forgiveness is described in many studies as a “buffer” for the negative impacts of victimization, mainly. The absence of self-regulation, in turn, can make aggressors more vulnerable to mental health problems. Similarly, this absence inhibits pro-victim or defensive behaviors when other students witness the aggressions.

Furthermore, in the results, it is perceived that the potential of the three character strengths in relation to bullying and cyberbullying is not directly associated with the emission of violent behaviors. The associations are with improved well-being, self-esteem, increased life or school satisfaction, and the mitigation of mental health problems. From a comprehensive perspective, gratitude, forgiveness, and self-regulation can favor the adoption of pro-social behavior. This, indirectly, can reduce the occurrence of peer violence. The positive trend revealed indicates that as students become more willing to demonstrate gratitude, forgiveness, and self-regulation, there is less propensity to engage in bullying/cyberbullying behaviors. At the same time, these values in action can also reduce the negative impact of victimization.

These findings may have significant implications for interventions aimed at promoting positive attitudes and behaviors among adolescents. Promoting gratitude, forgiveness, and self-regulation could be an effective strategy to create healthier environments and, consequently, reduce relationship problems or harmful behaviors in adolescence. In this sense, according to the reviewed studies, anti-bullying interventions should consider the lived experiences of students and implement activities that favor the reconnection of students with their strengths.

## 4. Discussion

These findings underscore the importance of promoting gratitude as a potentially effective tool in preventing and mitigating the negative impacts of bullying and cyberbullying on adolescents’ lives. In summary, the results highlight the role of forgiveness as a key factor in promoting emotional well-being and coping with challenges associated with bullying and cyberbullying in adolescence. Adolescents who reported more forgiveness, gratitude, and self-control were less likely to engage in aggression, and this should be considered in intervention programs.

Consistent with these findings from this review, other research on diverse themes has shown that presenting character strengths is a protective factor that ensures greater well-being and happiness, and reduces depressive symptoms [28,29]. Developing character strengths also facilitates overcoming traumatic events and maintaining positive emotions or moods [9,29,30]. In the case of bullying victims, according to the reviewed studies, expressing feelings of gratitude and forgiveness can decrease the negative impacts of victimization experiences. Improvements in mental health indices and a decrease in depressive symptoms were among the most significant data.

Gratitude has a beneficial effect, especially when evaluating subjective issues [28]. A study with two independent samples of Israeli adolescents (total 505 participants) also revealed that expressing feelings of gratitude facilitated the emission of pro-social behaviors and increased peer acceptance [6]. A systematic review that included 74 randomized clinical trials demonstrated that individuals subjected to gratitude-focused interventions improved their mental health and had fewer symptoms of anxiety and depression [29]. It is suggested that gratitude can play a protective role against risk behaviors and health problems.

Undoubtedly, the most intriguing findings of this review involve the approach to forgiveness. While not advocating that victims should forgive their tormentors, the exercise of forgiveness seems to represent a less ambiguous behavioral response than gratitude and is more clearly related to other psychological phenomena. Furthermore, forgiveness may require more time to develop as the person regulates their aversive emotions (e.g., anger) and intentions of revenge or retaliation [8].

A remarkably similar pattern of findings suggested that forgiveness is related to better interpersonal relationships and more social engagement based on desirable social values for maintaining social order [9]. Additionally, individuals with a greater willingness to forgive others tend to be physically healthier [5,9]. Forgiveness is also essential for developing a sense of trust in relationships, and for bullying/cyberbullying victims this can be crucial, as victims tend to establish patterns of mistrust when in relationships with others, and this aspect extends to other life stages.

On the other hand, self-regulation tends to prevent mental health problems [28]. Being a feeling that manifests more objectively in behavior, related to the ability to control impulses, regulate emotions, and maintain healthy behaviors, students with good self-regulation can manage the stress experienced in schools when involved in bullying/cyberbullying situations and can seek help when necessary.

It is also important to mention that studies on character strengths indicate that, even when experiencing adversities in childhood or adolescence (such as bullying or cyberbullying), possessing characteristics such as gratitude and self-regulation favors the maintenance of mental health [5,30]. Character strengths can be considered as personal resources that contribute to well-being and quality of life, highlighting the importance of addressing not only external factors in anti-bullying interventions but also strengthening the positive personality traits of students.

The reviewed data also have practical implications. To effectively implement programs that nurture gratitude, forgiveness, and self-regulation among students, schools can adopt a variety of structured activities. For example, gratitude journals can be introduced where students regularly write about things they are thankful for, fostering a positive mindset and enhancing emotional well-being. Schools can also organize workshops and role-playing activities that teach students the importance of forgiveness, helping them to understand and practice resolving conflicts peacefully. Furthermore, self-regulation skills can be developed through mindfulness sessions and stress management techniques, enabling students to better control their impulses and emotions. Storytelling sessions where students discuss characters’ actions in books or historical events can also highlight the importance of these virtues. These strategies not only promote a healthier school environment, but also equip students with essential life skills, contributing to their overall development and reducing incidences of bullying and cyberbullying.

Finally, although this review has many strengths, its results should be interpreted considering its three main limitations. Firstly, the reviewed studies are limited to seven countries, and conclusions about the effects of gratitude, forgiveness, and self-regulation in bullying and cyberbullying situations cannot be generalized to different countries and cultures. Secondly, the psychological well-being results measured in the included studies were not the same since the measurement tools used were not identical. Additionally, this review focuses on personal character strengths, and other variables that contribute to the occurrence of bullying/cyberbullying, such as social and contextual factors, were not considered. The review also considered the situation in different countries, but intercultural differences were not considered in the data analysis. This could be the subject of further investigation. Despite these limitations, this work broadens the perspective on phenomena that affect the health and development of students, contributing to the current body of knowledge produced.

## 5. Conclusions

In summary, the conclusions of this study provide some evidence about the potential of character strengths to promote individual well-being, especially when analyzing painful experiences of bullying or cyberbullying in adolescence. Other researchers are encouraged to leverage the findings of this review in the context of schools in different countries and cultures. Empirical research evaluating the presented findings could be useful for psychologists and may capture culturally sensitive aspects that were not identified in the reviewed studies, for example.

## Figures and Tables

**Table 1:** Search strategies applied in databases.

Databases	Strategies
Web of Science	Cross-terms: gratitude AND bullying; forgiveness AND bullying; self-regulation AND bullying. Limits: All Fields; Publication Years: 2023 OR 2022 OR 2021 OR 2020 OR 2019; Document Types: Articles; Languages: English [there were no texts in other languages].
Scopus	Cross-terms: gratitude AND bullying; forgiveness AND bullying; self-regulation AND bullying. Limits: All Fields; Publication Years: 2023 OR 2022 OR 2021 OR 2020 OR 2019; Document Types: Articles; Languages: English OR Spanish OR Portuguese.
Scielo	Cross-terms [in Portuguese]: gratidão AND bullying; perdão AND bullying; autorregulação AND bullying. Limits: All Files; By Subject; No Time Limitations.

**Table 2:** Identification and characteristics of the reviewed corpus.

Reference	Country	Objective	Variables of Interest	Participants/Samples	Main Results
[15]	Spain	Examine the relationships between gratitude, emotional intelligence, and cyber aggression.	Gratitude, emotional intelligence, and cyberbullying	1157 adolescents (girls = 54.4%) aged between 12 and 18 years (average age 13.78 years; SD = 1.33)	The findings highlight significant and negative correlations between cyber aggression and gratitude, as well as dimensions of emotional intelligence. Gratitude and emotional intelligence dimensions also show positive correlations. The results strongly support gratitude’s role as a significant mediator in the relationship between emotional intelligence dimensions and cyber aggression, explaining partially or entirely how emotional intelligence dimensions affect cyber aggression behavior.
[16]	China	Investigate the roles of mindfulness and gratitude in the relationship between cyberbullying perpetration and depression among children and adolescents.	Gratitude, mindfulness, and cyberbullying	1298 students (boys = 51.2%) aged between 9 and 16 years (average age 13.57 years; SD = 1.27)	Practicing cyberbullying is associated with increased depression levels, and students engaging in cyberbullying are less likely to be attentive and mindful of their actions and thoughts in the present moment (lower mindfulness). Gratitude or feeling thankful does not influence a student’s involvement in cyberbullying. Mindfulness and gratitude mediate the perpetration of cyberbullying and depression.
[17]	Turkey	Analyze how forgiveness and coping behaviors in cyberbullying function together as two mediators in a series of mediation models in a sample of Turkish adolescents.	Forgiveness, well-being, and cyberbullying	337 adolescents (boys = 50.1%) aged between 14 and 19 years (average age = 16.56 years).	There is a significant negative relationship between cyber victimization and well-being: as cyber victimization increases, students’ well-being decreases. A moderate, positive, and significant relationship exists between well-being and forgiveness, indicating that higher forgiveness levels are associated with better well-being. Adolescents who reported being able to forgive or had effective strategies to deal with cyberbullying showed higher well-being despite victimization.
[18]	Spain	Study the relationship between character strengths (forgiveness and gratitude), happiness, and pro-social behavior in bullying bystanders.	Forgiveness, gratitude, happiness, pro-social behavior, and bullying	1000 adolescents aged between 12 and 18 years (average age 14.70; SD = 1.58)	A significant positive correlation is found between gratitude, forgiveness, happiness, and pro-social behavior in bullying. Girls tend to report higher levels of gratitude, forgiveness, happiness, and pro-social behavior. According to an alternative statistical model, pro-social behavior positively influenced happiness and gratitude but not forgiveness.
[19]	Mexico	Examine the direct and mediating relationships between forgiveness, gratitude, self-control, and proactive and reactive aggression in bullying.	Forgiveness, gratitude, self-control, and bullying	1000 adolescents aged between 12 and 17 years	Significant negative correlations were found between forgiveness, gratitude, self-control, and proactive and reactive aggression. Adolescents reporting higher levels of forgiveness, gratitude, and self-control were less likely to engage in proactive and reactive aggression. Boys demonstrated more reactive and proactive aggression and less gratitude than girls.
[20]	Spain	Analyze the relationships between forgiveness, revenge motivations, avoidance, and benevolence; loneliness and subjective evaluations of social networks; and relational, physical, or verbal victimization based on gender.	Forgiveness, revenge motivations, avoidance, benevolence, loneliness, social network, and victimization	617 adolescents (50.7% = boys) aged between 10 and 16 years (average age 13.04 years; SD = 1.80)	Adolescents with high levels of victimization showed a greater desire for revenge, increased avoidance motivation, higher emotional loneliness, and a more negative subjective evaluation of their social networks. Forgiveness-related characteristics, like the willingness to forgive or not, had a significant impact on adolescents’ experiences with peer victimization.
[21]	China	Test a moderated mediation model of forgiveness and self-esteem concerning the association between peer victimization and subjective well-being.	Forgiveness, self-esteem, well-being, and victimization	2758 adolescents aged between 10 and 19 years (average age 13.53 years; SD = 1.06)	The interaction between peer victimization and forgiveness in self-esteem was significant. For adolescents with low forgiveness (reactive), victimization had a significantly negative impact on self-esteem. For adolescents with high forgiveness (protective), the negative effect of victimization on self-esteem was even stronger. Regardless of forgiveness levels, peer victimization had a similar impact on subjective well-being.
[14]	Peru	Evaluate, longitudinally, the effect of experiencing cybervictimization and other forms of traditional bullying simultaneously on life and school satisfaction.	Gratitude, life and school satisfaction, cyberbullying, and bullying	221 adolescents; average age: 12.09 years (SD = 0.89)	Victims of cyberbullying who also experienced other forms of traditional bullying were more likely to have lower life satisfaction and school satisfaction compared to those who did not experience aggression. Students with high levels of gratitude maintained stable levels of life satisfaction, regardless of cyberbullying prevalence.
[22]	Peru	Examine the roles of dispositional and situational moral emotions in bullying and pro-social behavior in adolescents.	Gratitude, compassion, moral development, pro-social behavior, and bullying	Two studies: 644 adolescents aged between 14 and 18 years (average age 15.6 years; SD = 1.4); 235 adolescents aged between 10 and 15 years (average age 12.5 years; SD = 0.9)	Gratitude and compassion were positively related to pro-social behavior towards victims, and adolescents more sensitive to acts of kindness tended to engage less in bullying behaviors. There was a statistically significant trend of increased willingness in adolescents to express gratitude and engage in pro-social behaviors.
[23]	Spain	Investigate the mediating roles of stress and lack of forgiveness in the link between cybervictimization and cyberbullying aggression.	Forgiveness, stress, and cyberbullying	979 adolescents (girls = 55.4%) aged between 12 and 18 years (average age 13.72; SD = 1.31)	Cybervictimization was significantly associated with stress and revenge motivation (less forgiveness). Cybervictimization did not lead adolescents to engage in avoidance behaviors (forgiveness) as a response.
[24]	Spain	Explore the relationships between bullying victimization, gratitude, and suicide risk in a sample of adolescents; explore gender differences in the association between variables; determine if there is a significant interaction effect of victimization × gratitude in predicting suicide risk.	Gratitude, suicide, depression, and bullying victimization	1617 adolescents (girls = 50.5%) aged between 12 and 17 years (average age 14.02 years; SD = 1.46)	Adolescents who were victims of bullying were more likely to experience depression symptoms or think/act suicidally. Victimized students tended to have fewer feelings of gratitude. Participants who reported higher gratitude had fewer depression symptoms and a lower likelihood of suicidal thoughts/behaviors. Girls who were victims of bullying and also had high levels of gratitude tended to experience fewer depressive symptoms compared to those with low levels of gratitude.
[25]	Italy	Examine the mediating effect of dispositional forgiveness on the relationship between cybervictimization and cyberbullying, and explore the moderating effect of gender in this relationship.	Forgiveness and cyberbullying	481 adolescents (boys = 52.4%) aged between 14 and 19 years (average age 17.2 years; SD = 1.5)	Girls reported higher levels of forgiveness and cybervictimization than boys, but both groups showed comparable levels of involvement in cyberbullying. Victimization has a negative influence on forgiveness disposition: as cybervictimization increases, the willingness to forgive decreases. Dispositional forgiveness negatively influences cyberbullying behavior, meaning higher forgiveness levels are associated with a lower likelihood of involvement in cyberbullying. Although cybervictimization is related to a lower willingness to forgive in both genders, this effect is more pronounced in male adolescents.
[26]	Mexico	Investigate the relationship between guilt, sympathy, and aggressive defense intervention in cyberbullying situations; analyze how self-regulation mediates the influence of moral emotions on intervention behaviors.	Self-regulation, moral emotions, and cyberbullying	1674 adolescents (girls = 50.3%); average age of boys = 15.99 years (SD = 1.03); average age of girls = 16.02 years (SD = 1.04)	Analyses revealed that adolescents rarely expressed feelings of guilt and sympathy in cyberbullying events, demonstrating moderate self-control but rarely intervening to defend victims. Being female was positively related to self-control. The negative correlation between self-control and aggressive defense behavior suggests that the better someone is at self-regulating their emotions and actions, the less likely they are to adopt an aggressive intervention when witnessing cyberbullying.
[27]	Australia	Determine the effects of rewriting forgiveness or revenge images in a bullying victimization scenario.	Forgiveness, revenge, and bullying	43 boys aged between 12 and 14 years (average age 12.81 years; SD = 0.70)	Most participants reported experiencing some form of bullying in the previous semester, with verbal bullying being the most common. Participants considered the aggressive event less serious over time (forgiveness).

## Data Availability

Primary and Supplementary materials and tables can be requested from the authors.

## Supplementary Materials

The following supporting information can be downloaded at: https://www.mdpi.com/article/10.3390/ijerph21070839/s1, Table S1. Data collection and analysis characteristics of the reviewed studies.
