# Supplementary material for: Bullying and Cyberbullying in School: Rapid Review on the Roles of Gratitude, Forgiveness, and Self-Regulation
Source: Int J Environ Res Public Health. 2024 Jun 27;21(7):839. doi: 10.3390/ijerph21070839 (PMC11276976; doi:10.3390/ijerph21070839)
Supplement: Supplementary file 1 [file ijerph-21-00839-s001.zip › ijerph-3014494-supplementary.pdf]

Table S1

Características de coleta e análise de dados dos estudos revisados.

Referência	Instrumentos	Mecanismos de validade e precisão
Chamizo-Nieto et al. 2020	Gratidão = Gratitude Questionnaire<br>Inteligência Emocional = Wong and Law's Emotional Intelligence Scale (WLEIS)<br>Comportamentos ciberagressivos = European Cyberbullying Intervention Project Questionnaire (ECIPQ)	Estatística descritiva, correlação de Pearson, modelo estrutural (critérios estatísticos foram aplicados para avaliar a qualidade do ajuste do modelo, qui-quadrado/grau de liberdade ($\chi 2/df$), raiz do erro quadrático médio de aproximação (RMSEA) e raiz padronizada), valores de SRMR inferiores a 0,08 indicam um ajuste adequado, e valores de índice de ajuste comparativo (CFI) de 0,95 ou superiores refletem um bom ajuste.
Chen & Zhu, 2022	Experiências de cyberbullying = versão chinesa do *European Cyberbullying Intervention Project Questionnaire* (ECIPQ)<br>Depressão = versão chinesa do *Beck Depression Inventory-II* (BDI-II)<br>Atenção plena = Medida de Atenção Plena para Crianças e Adolescentes<br>Gratidão = Questionário de Gratidão	Testes t e testes qui-quadrado; matriz de correlação; modelo SPSS PROCESS 59; variáveis de confusão foram controladas; nível de significância estatística fixado em 0,05.
Eroglu et al., 2022	Experiências de cyberbullying = Revised cyberbullying inventory (RCI)<br>Bem-estar = The five-dimensional scale of well-being for adolescents (EPOCH)<br>Perdão = Forgiveness scale for adolescents<br>Enfrentamento do cyberbullying = Scale for coping with adolescents' cyberbullying (SCAC)	Estatística descritiva, correlação de Pearson, modelos de equação estrutural
García-Vázquez et al., 2020a	Gratidão = Gratitude Questionnaire<br>Perdão = Forgiveness Heartland Scale<br>Felicidade = The scale of Orientation to Happiness<br>Comportamento pró-social = Subscale Prosocial Bystander	Estatística descritiva, análises univariadas, análises fatoriais confirmatórias (AFC), modelo de equações estruturais (SEM), medidas de invariância , teste qui-quadrado, SRMR, TLI, AGFI, CFI, RMSEA, bootstrap
García-Vázquez et al., 2020b	Gratidão = Gratitude Questionnaire<br>Perdão = Forgiveness Heartland Scale<br>Autocontrole = Short Self-Control Scale<br>Agressão reativa e proativa = Reactive-Proactive Aggression Questionnaire	Estatística descritiva, correlações de Pearson, modelos CFA e estruturais utilizando estimativa de máxima verossimilhança e bootstraps de intervalo de confiança corrigidos pelo viés (com 500 repetições e intervalo de confiança de 95%), Qui-quadrado, análise multigrupo
León-Moreno et al., 2021	Perdão = Transgression-Related Interpersonal Motivations Inventory (TRIM-18)<br>Solidão = Russell's Loneliness Scale	Estatística descritiva, Análise Fatorial Confirmatória (AFC), estimação de máxima verossimilhança e teste qui-quadrado, validade convergente, alfa de Cronbach (confiabilidade), ANOVA, MANOVA, testes univariados (ANOVAs) e teste post hoc de Bonferroni, regressão linear múltipla Stepwise

	Experiências de vitimização = The School Victimization Scale	
Liu et al., 2023	Questionário sociodemográfico<br>Experiências de Vitimização = Adolescent Peer Victimization Questionnaire<br>Bem-estar = Self-Esteem Scale<br>Bem-estar subjetivo = Subjective Well-Being Scale<br>Perdão = Forgiveness Scale	Testes t, análises de regressão, análises de variância confirmatória (CFA) e o método de bootstrap para realizar as análises de mediação e moderação e testar os modelos de relação entre variáveis no estudo
Oriol et al., 2021	Satisfação com a Vida = Satisfaction with Life Scale for Children<br>Satisfação Escolar = School Satisfaction<br>Vitimização por Cyberbullying = National Survey of School Violence<br>Vitimização por Bullying = National Survey of School Violence by MINEDU<br>Gratidão = Dispositional Gratitude	Análise fatorial confirmatória sendo empregado estimador robusto de máxima verossimilhança e índices de ajuste, para calcular a confiabilidade foram utilizados o alfa de Cronbach (α) e o ômega de McDonald (Ω), t-Student, Qui-quadrado, ANOVA, correlação de Pearson, índice de confiança de 95%
Oriol et al., 2023	Compaixão = Dispositional Compassion<br>Gratidão = Dispositional Gratitude<br>Bullying = Traditional Bullying Behavior<br>Comportamento pró-social em relação às vítimas = Prosocial Behavior Toward Victims<br>Moralidade = Moral Elevation during Bullying Situations Scale (MEBSC)	Análise fatorial confirmatória sendo empregado estimador robusto de máxima verossimilhança e índices de ajuste, para calcular a confiabilidade foram utilizados o alfa de Cronbach (α) e o ômega de McDonald (Ω), t-Student, Qui-quadrado, ANOVA, correlação de Pearson, índice de confiança de 95%
Quintana-Orts et al., 2020	Questionário sociodemográfico<br>Experiências de cyberbullying = European Cyberbullying Intervention Project Questionnaire was used (ECIP-Q)<br>Estresse = Depression, Anxiety and Stress Scales (DASS-21)<br>Perdão = Transgression-related Interpersonal Motivations-18 Scale (TRIM-18)	Imputação de dados, estatísticas descritivas, análises de correlação, modelo de mediação múltipla serial, macro PROCESS, correção de heterocedasticidade e não normalidade, variáveis de controle (covariáveis)
Rey et al., 2019	Vitimização por bullying = Victimization subscale of the European Bullying Intervention Project Questionnaire (EBIP-Q)<br>Risco de suicídio = Depression Inventory Short Version (CDI-S); Suicidal Behaviors Questionnaire–Revised (SBQ-R)<br>Gratidão = Gratitude Questionnaire	Estatística descritiva, correlação de Pearson, teste t de Student, análises de moderação, a significância do efeito indireto em diferentes níveis foi testada usando intervalos de confiança de bootstrap corrigidos pelo viés de 95%
Sechi et al., 2023	Experiências de cyberbullying = Florence Cyber-Bullying – Cyber-Victimisation Scales (FCBVSs)	Estatística descritiva, correlação de Pearson, testes t de amostras independentes, modelos de equações estruturais (SEM) testados por estimativa de máxima

	Perdão = Heartland Forgiveness Scale (HFS)	verossimilhança e a qualidade do ajuste dos modelos foi avaliada usando o índice de ajuste comparativo (CFI), o índice Tucker Lewis (TLI), a raiz quadrada média do erro de aproximação (RMSEA) e a raiz quadrada média residual padronizada (SRMR), análise de múltiplos grupos com AMOS, teste qui-quadrado
Valdés-Cuervo et al., 2021	Simpatia = Sympathy Scale<br>Culpa = adaptação da Moral Emotions Scale<br>Autorregulação = Self-Regulation Scale<br>Comportamento dos obervadores = Cyber Intervening Behavior Scale	Estatísticas descritivas, análise de correlação, testes t, análise fatorial confirmatória (AFC), modelos de equações estruturais (SEM), estimação de máxima verossimilhança, bootstrap de Bollen-Stine, não normalidade multivariada, índices de ajuste do modelo, efeitos indiretos (bootstrapping)
Watson et al., 2021	Saúde mental = 21-item Depression Anxiety Stress Scales (DASS 21)<br>Experiências de bullying = perguntou-se aos participantes com que frequência eles haviam sofrido bullying físico, verbal ou on-line<br>Afetos positivos e negativos = Positive and Negative Affect Schedule – Child (PANAS-C)<br>Respostas avaliativas = os participantes lembravam uma situação passada de bullying e eram provocados a visualizarem um fim de perdão ou vingança	Estatística descritiva, teste de Mann-Whitney, ANOVA, análise de componentes
